# A Two-Phase System for the In Vitro Culture of *Agave guiengola* Gentry

**DOI:** 10.3390/plants15111653

**Published:** 2026-05-28

**Authors:** Iván Maldonado-Zavala, José Juvencio Castañeda-Nava, Lourdes Delgado-Aceves, José Manuel Rodríguez-Domínguez, Benjamín Rodríguez-Garay, Antonia Gutiérrez-Mora

**Affiliations:** Centro de Investigación y Asistencia en Tecnología y Diseño del Estado de Jalisco, A.C., Unidad de Biotecnología Vegetal, Zapopan 45019, Jalisco, Mexico; ivmaldonado_al@ciatej.edu.mx (I.M.-Z.); jcastaneda@ciatej.mx (J.J.C.-N.); madelgado_pos@ciatej.edu.mx (L.D.-A.); mrodriguez@ciatej.mx (J.M.R.-D.); agavero01@hotmail.com (B.R.-G.)

**Keywords:** silver agave (*Agave guiengola* Gentry), micropropagation, culture medium, axillary bud proliferation, organogenesis, hyperhydricity

## Abstract

*Agave guiengola* Gentry is a microendemic ornamental species native to Oaxaca, México, with restricted distribution and threatened by human activities, placing it at risk of extinction. Two culture media were evaluated: Murashige and Skoog (MS) medium and modified MS medium, together with medium consistency (semisolid and liquid) and plant growth regulators (PGRs): 6-benzylaminopurine (BA) (5.0 mg L^−1^) for MS media and a combination of kinetin (KIN) and 3-indoleacetic acid (IAA) (5.0–9.0 and 0.1–1.0 mg L^−1^ respectively) for modified media. Shoot clusters were grown in two consecutive 30-day phases, with transfer to opposite consistency after the first month of cultivation (first phase). Growth, hyperhydricity, rooting and callus formation were evaluated. Treatments using modified formulation + KIN + IAA under both consistencies had the greatest growth (5.5 and 5.0 cm^2^). Hyperhydricity was more frequent in liquid MS media (up to 100%), while adjusted MS medium had lower incidence with or without PGRs (0.0% and 6.6%). Transfer from liquid-to-semisolid medium reduced hyperhydricity (10.47%) compared with the reverse (31.35%). Rooting occurred frequently with modified formulation media. Callogenesis occurred mainly using MS + BA media (up to 93.2%), associated with adventitious shoot formation. A synergistic biphasic effect is proposed using semisolid MS + BA followed by modified MS liquid + KIN + IAA, optimizing in vitro growth for conservation.

## 1. Introduction

*Agave* is a genus of monocotyledonous plants comprising more than 200 species widely distributed throughout the Americas, ranging from southern Canada to South America [1]. In México, 75% of the genus species are found throughout the country [2]. They are perennial plants, reproducing both sexually and asexually, with a fibrous, fleshy, and succulent structure [3], adapted to unfavorable conditions, such as arid environments with excessive solar radiation, high temperatures, and prolonged drought [4]. They play an important role in maintaining ecosystem balance, given their interactions with insects and higher animals, serving as pollinators as well as a source of food and shelter [3]. Historically and to the present day, agaves have played an important cultural and economic role in México, due to their diverse uses in food, distilling, fibers, and materials, as well as their potential biotechnological applications for biofuels and bioplastics, among others [5].

*Agave guiengola* Gentry (*A. guiengola*) is a microendemic species of México; it inhabits the Sierra Guiengola in the Tehuantepec District (Oaxaca). It has a limited distribution within the area and a small population. Its survival is constantly threatened by human activities, including mining in the area, predation, and illegal trade, which has been classified as an endangered species on the Red List of Species and the NOM-059-SEMARNAT-2010 [6,7,8]. Its uses are primarily ornamental; however, its potential has not been fully explored due to its status, making it a species of potential interest.

Micropropagation has proven to be an integral and efficient tool in strategies for the conservation and utilization of threatened species [9]. In *Agave*, the first in vitro propagation studies were conducted during the late 1970s and 1980s by Groenewald et al. [10], Robert et al. [11], and Powers and Backhaus [12]. Since then, successful propagation protocols have been developed for several species, including *A. sisalana* [13], *A. tequilana* [14,15], *A. salmiana* [16], *A. cupreata*, *A. difformis*, *A. obscura*, *A. potatorum* [17] and *A. americana* [18]. In vitro studies on *Agave* have reported the use of different culture media and plant growth regulators (PGRs) [19]. In addition, in recent years, the propagation of *A. maximiliana* via axillary bud proliferation has been reported [20], while de la Cruz-Olvera et al. [21] developed a micropropagation protocol for *A. angustifolia*. Likewise, Aguilar-Jiménez et al. [22] developed an in vitro conservation protocol for *A. potatorum* and *A. marmorata* based on a slow-growth culture medium, which enabled the plants to remain viable, preserve their regenerative capacity, and maintain their original morphological characteristics for more than 24 months, contributing to germplasm conservation. Rescalvo-Morales et al. [23] reported on the micropropagation of *A. cupreata* via organogenesis and the influence of the mother plant’s age on its in vitro propagation. For *A. guiengola*, information is limited; Vargas [24] induced indirect organogenesis using stem base explants and 0.5 mg L^−1^ of 1-naphthaleneacetic acid (NAA) and 2.0 mg L^−1^ of 6-benzylaminopurine (BA).

In recent years, liquid culture systems have gained increasing attention over traditional semisolid media because they provide greater availability of nutrients and PGRs, improved aeration, and consequently, higher propagation rates and accelerated growth of higher-quality plant material [25]. However, to fully exploit these advantages, it is often necessary to use temporary immersion systems (TISs) to ensure efficient aeration and prevent problems such as hyperhydricity and chlorosis. The use of TISs has been reported in several *Agave* species, including *A. angustifolia* [26], *A. tequilana* [27], *Agave* spp. [28]; *A. marmorata* [29] and *A. potatorum* [30]. Similarly, Chávez-Ortíz et al. [31] propagated *A. guiengola* using axillary buds cultured in semisolid medium and TISs, obtaining up to 3.7 shoots per explant in semisolid medium supplemented with 2.0 mg L^−1^ of BA. These authors also reported high multiplication efficiencies when shoot clusters were used as explants in TIS-based propagation systems. Therefore, the present study aimed to evaluate the effects of two different culture media, medium consistency, and PGRs in a two-phase culture system on the in vitro development and morphophysiological responses of *A. guiengola*.

## 2. Materials and Methods

### 2.1. Plant Material

The explants used consisted of in vitro-cultured clusters of *A. guiengola* shoots, previously obtained through axillary bud propagation on MS medium [32] supplemented with 5.0 mg L^−1^ BA, based on preliminary assays conducted at the Unidad de Biotecnología Vegetal of the Centro de Investigación y Asistencia en Tecnología y Diseño del Estado de Jalisco, A.C. (CIATEJ), Zapopan, Jalisco, México (unpublished data) as well as the available literature for *Agave* species [33,34,35]. The plant material was obtained from the germplasm collection of the research center.

### 2.2. Effect of the Two-Phase Culture System on the In Vitro Cultivation of A. guiengola

Two culture media with different consistencies (semisolid and liquid) and different PGR combinations were evaluated as treatments (Table 1). The culture media evaluated were formulated as follows: the first medium (M1) consisted of a basal formulation of MS salts [32], supplemented with L2 vitamins [36] and, when applicable, with 5.0 mg L^−1^ BA (Sigma-Aldrich^®^, St. Louis, MO, USA) (T3–T4). The second medium (M2) was based on a modified Murashige and Skoog (MS) formulation. This modification involved increasing the concentrations of potassium nitrate (KNO_3_) and calcium chloride (CaCl_2_·H_2_O) and reducing the concentration of ammonium nitrate (NH_4_NO_3_). The concentration of potassium nitrate was adjusted to a range of 2.0–5.0 g L^−1^; that of ammonium nitrate to 0.1–1.0 g L^−1^ and that of calcium chloride to 0.5–1.0 g L^−1^. The M2 medium for some treatments (T7–T8) was supplemented with the cytokinin kinetin (KIN) at a concentration of 5.0–9.0 mg L^−1^ and the auxin 3-indoleacetic acid (IAA) at 0.1–1.0 mg L^−1^. Both media were prepared in two consistencies: semisolid, gelled with 8.0 g L^−1^ agar (Sigma-Aldrich^®^), and liquid, without gelling agents. *A. guiengola* explants were excised from shoot clusters and cut into cluster segments approximately 1.0 cm^2^. Explants were cultured in 140.0 mL glass jars (12.0 × 4.5 cm) containing 25.0 mL of culture medium for semisolid treatments and 5.0–12.0 mL for liquid treatments.

Explants were cultured for 30 d (first phase), after which growth was evaluated based on explant size (area, cm^2^) using a millimeter ruler. Hyperhydricity was recorded according to visual characteristics associated with the disorder, including swollen tissues caused by excessive water accumulation and a translucent or glassy appearance. Root and callus formation were also evaluated. Subsequently, explants were transferred to fresh medium with the same formulation but the opposite consistency and cultured for an additional 30 d (second phase) before a second evaluation. All media were supplemented with 30.0 g L^−1^ sucrose, adjusted to pH 5.8 ± 0.02, and autoclaved at 121 °C and 1.27 kg cm^−2^ for 15 min. Cultures were maintained in an incubator at 27 ± 2 °C under a 16 h light/8 h dark photoperiod (LED lighting, 25 µmol m^−2^ s^−1^).

### 2.3. Statistical Analysis

Eight independent treatments were evaluated, with five replicates per treatment and three explants per replicate. A completely randomized experimental design was used to evaluate explant growth (area, cm^2^) and the percentage of explants exhibiting hyperhydricity, root formation, and callus production. Data were analyzed using Statgraphics Centurion^®^ XV statistical software 15.2.06 (StatPoint Technologies, Inc., Warrenton, VA, USA) through a one-way analysis of variance (ANOVA), after removing outliers when necessary. Mean comparisons were performed using Tukey’s HSD test at a significance level of *p* < 0.05. Explant area was considered a continuous quantitative variable, whereas hyperhydricity, root formation, and callus production were initially recorded as categorical (qualitative) variables and subsequently converted into percentages for quantitative statistical analysis.

## 3. Results

### 3.1. Growth of Explants

**Figure 1:**
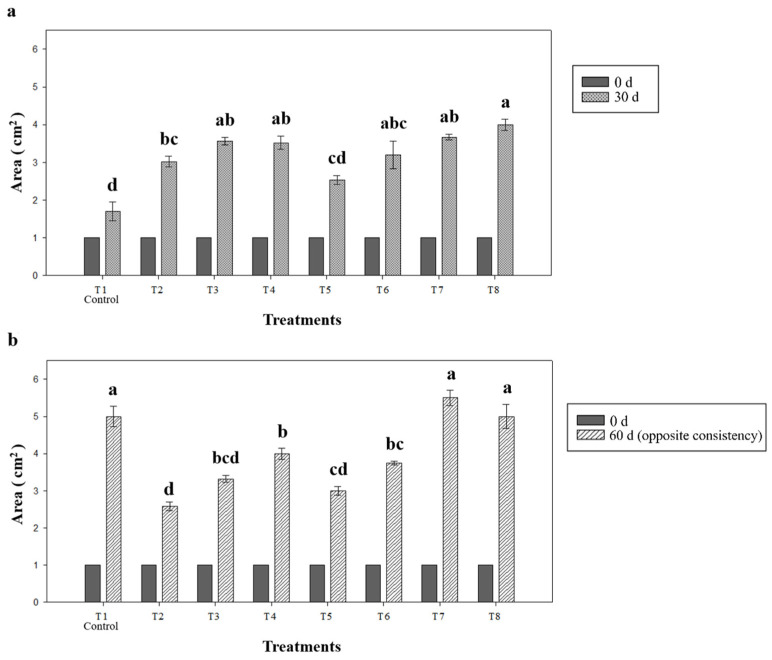
Mean explant sizes (area in cm^2^) of *A. guiengola* Gentry at: (**a**) 30 d of cultivation (first phase) and (**b**) 60 d of cultivation (second phase using the same medium, but with the opposite consistency). The line above each bar represents the standard error. Different letters between bars indicate significant differences according to Tukey’s HSD test (*p* < 0.05). d = day; T = treatment.

**Figure 2:**
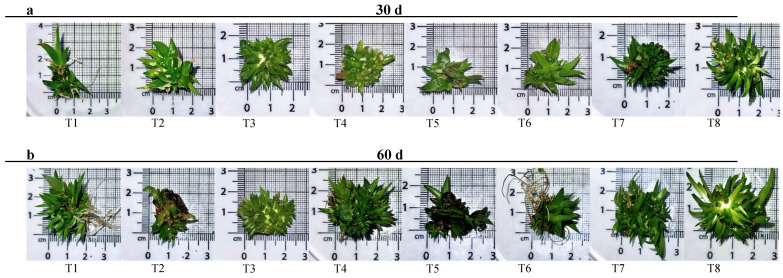
Effect of culture medium, consistency, and PGRs in the two-phase culture system for the in vitro propagation of *A. guiengola*. From left to right: treatments T1–T8. (**a**): at 30 d of cultivation (first phase) and (**b**): at 60 d of cultivation (second phase using the same medium, but with the opposite consistency). (**a**): T1: explant with development of some shoots and presence of roots; T2: formation of small calli on the leaf surface; T3: explant generating poorly differentiated shoots; T4: hyperhydric explant; T5–T7: explant with some differentiated shoots; T8: explant with multiple differentiated and developed shoots. (**b**): T1: explant with shoots and roots; T2: explant with oxidized tissue; T3–T4: explant with multiple poorly differentiated shoots; T5: explant with some differentiated shoots; T6: explant with high root production; T7–T8: explant with multiple fully developed and differentiated shoots. d = day; T = treatment.

**Figure 3:**
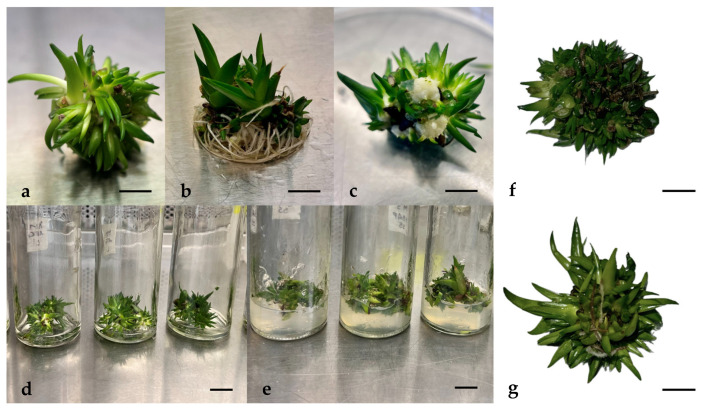
In vitro culture of *A. guiengola*. (**a**) cluster of shoots at 30 d of cultivation (T8). Scale bar = 1 cm; (**b**) explants with high root production at 60 d after sowing (T6). Scale bar = 1 cm; (**c**) explant with organogenic callus developing adventitious shoots at 50 d of cultivation (T3). Scale bar = 1 cm; (**d**,**e**) culture methods: (**d**) culture in semisolid medium and (**e**) culture in liquid medium. Scales bar = 2 cm; (**f**,**g**) Explant cultures: (**f)** M1 + 5.0 mg L^−1^ BA SS at 30 d post-culture and (**g**) Explant cultured in a two-phase medium: M1 + 5.0 mg L^−1^ BA SS → M2 + KIN + IAA L (60 d of cultivation). Scale bar = 1 cm; T = treatment; SS = semisolid consistency; L = liquid consistency; → = change in consistency.

After 30 d of culture, all treatments promoted explant growth (Figure 1a). The greatest growth was observed in the modified medium (M2) supplemented with KIN + IAA (T7 and T8), with 5.5 and 5.0 cm^2^, respectively, and these results persisted until the end of the experiment (Figure 1b). In contrast, the control treatment cultured in MS (M1) without plant growth regulators under a semisolid-to-liquid transition (T1 control) showed the lowest growth during the first evaluation (1.70 cm^2^). Meanwhile, M1 media supplemented with BA (T3 and T4) also exhibited considerable growth, with values close to those of M2 with KIN + IAA at 30 d; however, during the second phase of the experiment, their growth did not increase significantly; furthermore, the observed growth was mainly associated with morphological responses related to axillary bud proliferation (Figure 2).Modified medium (M2) with KIN + IAA and under liquid-to-semisolid transition (T8) promoted greater shoot development and differentiation in the explants compared to the other treatments (Figure 3a). In the case of T1, after 60 d of culture, it reached the same size as T8, at 5.0 cm^2^; however, this growth was attributed to hyperhydricity (Table 2). On the other hand, explants cultured in M1 medium without PGRs under a liquid-to-semisolid transition (T2), and those cultured in M1 medium supplemented with 5.0 mg L^−1^ BA under a semisolid-to-liquid sequence (T3) decreased in size by the end of the second phase. Furthermore, unlike the other treatments, some T2 explants showed signs of possible tissue oxidation, which was qualitatively assessed by visual inspection based on the darkening of the tissues to a brown or black color and a loss of turgor at the end of the culture (Figure 2b).

### 3.2. Percentage of Hyperhydric Explants

When evaluating the effects of treatments and culture medium composition on hyperhydricity, significant differences were observed (Table 2). During the first phase of the experiment, hyperhydricity occurred more frequently in treatments cultured in M1 medium, particularly in those supplemented with 5.0 mg L^−1^ BA and initially established in liquid medium before transfer to semisolid medium (T4), where 100% of the explants exhibited hyperhydricity. In contrast, treatments formulated with M2 medium showed a lower incidence of this disorder. The semisolid-to-liquid treatment without PGRs (T5) yielded the best response, with no explants exhibiting hyperhydricity. After completing the second phase (60 d), explants cultured in M1 medium with BA under a semisolid-to-liquid transition (T3), and those cultured in modified M2 medium with KIN + IAA under the same consistency sequence (T7) had the highest percentages of hyperhydricity (40% and 39.6%, respectively). Overall, a greater incidence persisted in cultures established in M1 medium than in those cultured in M2. In contrast, M2 treatments established under liquid-to-semisolid transitions, either without PGRs (T6) or with KIN + IAA (T8), had the lowest percentages throughout the experiment (6.6% and 0.0%, respectively).

### 3.3. Size and Hyperhydricity in a Two-Phase System Under Opposite Medium-Consistency Transitions

After 60 d of two-phase culture, the evaluated variables showed different responses depending on the direction of the medium consistency transition (Table 3). The sequence in which medium consistency was changed within the two-phase system did not produce statistically significant differences in explant growth, suggesting that the order of consistency transition had no direct effect on growth. In contrast, significant differences were observed in the percentage of hyperhydric explants between the two transition systems. The liquid-to-semisolid transition system showed a substantially lower percentage of hyperhydric explants (10.47%), whereas cultures transferred from semisolid-to-liquid medium showed a higher incidence (31.45%), representing nearly a threefold increase compared to the reverse transition system.

### 3.4. Root Production

Root formation occurred in most of the treatments, except in treatments cultured in M1 medium + 5.0 mg L^−1^ BA under both semisolid-to-liquid (T3) and liquid-to-semisolid (T4) transitions, which showed no root development throughout the entire growing period (0.0%). The highest rate of root development at 30 d was observed in explants cultured without PGRs in the M1 medium under a semisolid-to-liquid sequence (T1) and in the modified M2 medium under a liquid-to-semisolid sequence (T6) (100%) (Figure 2), a trend that persisted until the end of the experiment, even when the consistency of the culture medium was reversed; however, explants cultured in the modified M2 medium under the liquid-to-semisolid system (T6) showed greater rooting response (Figure 3b). Compared with treatments cultured in M1, all treatments using medium M2 showed rooting at the end of the study.

### 3.5. Callus Production

Callus formation at 30 d was most evident in explants cultured in M1 medium without PGRs under a liquid-to-semisolid transition (T2) and in M1 medium with 5.0 mg L^−1^ BA under the same liquid-to-semisolid system (T4). However, after 60 d of culture, explants cultured in M1 media + BA (T3 and T4) showed more frequent callus formation (59.8% and 93.2%, respectively), as well as the formation of adventitious shoots (Figure 3c). All other treatments except modified M2 medium without PGRs under a semisolid-to-liquid sequence (T5, 0.0%) also generated callus during the first or second phase of the experiment in some of the explants. However, this response was limited and localized, resulting in small calli barely visible on the leaf surface, which did not induce subsequent morphogenic responses. Furthermore, the analysis suggests that both liquid-consistency culture media (M1 and M2) favored callus formation on the young leaves of the explants. However, the expression of this response was transient and practically imperceptible after the first few weeks of culture.

## 4. Discussion

### 4.1. Growth of Explants

At the end of the first culture phase, growth was observed in explants from all treatments, with the greatest growth observed in explants cultured in M2 media containing KIN and IAA. The M2 medium has been previously evaluated in other *Agave* species and has been shown to promote plant growth (unpublished data). Similarly, modifications to the M2 medium that include an increase in potassium nitrate (KNO_3_) and a reduction in ammonium nitrate (NH_4_NO_3_) provide a better balance of NH_4_^+^ and NO_3_^−^ ions. Furthermore, the adjustment to the calcium (CaCl_2_) concentration enhances the structural and physiological properties of cell membranes and walls. All of the above results in accelerated explant growth and improved quality in the plants produced. In contrast, explants cultured in the control M1 medium without PGRs under a semisolid-to-liquid transition (T1) exhibited the lowest growth, likely due to the absence of stronger stimuli to induce cell division, multiplication, and development, as is the case with some of the PGRs [37] used in the other treatments. At the end of the second culture phase, explants from this control treatment reached sizes similar to those cultured in modified M2 medium with KIN + IAA under a liquid-to-semisolid sequence (T8); however, it was observed that this growth was influenced by hyperhydricity, an unfavorable physiological disorder for explant development characterized by the appearance of vitreous, fragile, and translucent tissues [38]. Some studies have documented similar effects when comparing semisolid and liquid media in other species [39,40] and in *Agave* [29,41]. On the other hand, the explants in M1 medium without PGRs under a liquid-to-semisolid transition (T2) and those cultured in M1 + 5.0 mg L^−1^ BA under a semisolid-to-liquid sequence (T3) reduced their size by the end of the second phase. Additionally, some explants cultured in M1 medium without PGRs under the liquid-to-semisolid system (T2) showed tissue oxidation; this response may have been associated with the change in medium consistency could cause stress in the explants, a potential increase in the production of reactive oxygen species (ROS), or accumulation of phenolic compounds [42]; in this study, none of these parameters were evaluated in greater detail; however, these phenomena have been observed in other studies [43,44,45]. In cultures maintained in liquid medium, the explant surface is in direct contact with the culture medium, leading to the release of toxic metabolites that accumulate in the tissue area and disperse more effectively in liquid than in semisolid medium. A similar effect may have occurred in the present study when explants were transferred from liquid to semisolid medium.

Although direct propagation could not be quantified in the present study because the explants consisted of clusters of underdeveloped shoots, explant growth was evaluated as an indirect response. Furthermore, the use of BA to promote propagation via axillary bud proliferation has been widely reported [46,47,48], as has its role in inducing organogenesis [34,49,50]. These morphogenic responses were also clearly observed in the present study. Similarly, Chávez-Ortíz et al. [31] reported comparable responses in *A. guiengola* when evaluating the effects of cytokinins BA and 6-(γ-γ-dimethylallylamino) purine (2iP) on propagation. Those authors observed that 2iP induced larger and better-differentiated shoots, although with lower propagation rates than BA, which more frequently produced compact clusters of poorly differentiated shoots. In such cases, the shoots were too small to separate and quantify individually; however, the use of isolated shoots as explants allowed propagation to be evaluated more accurately. These observations suggest that a similar response may have occurred in the present study.

In subsequent tests, good results were obtained by combining both media and their respective PGRs in a two-phase culture. During the first phase, culturing explants in semisolid M1 medium supplemented with 5.0 mg L^−1^ of BA promoted the proliferation of multiple axillary shoots (Figure 3f). Subsequently, transfer to liquid M2 medium with KIN and IAA during the second phase maximized the growth and differentiation of the shoots from the clusters (Figure 3g), resulting in an efficient cultivation process. Regarding the methodology previously reported for *A. guiengola* [31], in which BA and 2iP were evaluated for shoot proliferation in semisolid MS medium, some BA-containing treatments did not produce well-developed individual shoots, but rather compact clusters of small and poorly differentiated shoots. Consequently, these propagules could not be separated for rooting, making additional culture stages necessary to achieve complete shoot growth and differentiation. These stages included the use of temporary immersion systems (TISs), followed by cultivation in semisolid MS medium without growth regulators to induce rooting. As a result, the complete process required a prolonged cultivation period, including approximately two months for proliferation induction, two to three additional months for shoot differentiation using TISs, and another two months for rooting and shoot growth before acclimatization. In contrast, the methodology proposed in the present study improves and simplifies the in vitro cultivation process of *A. guiengola*. The two-phase system, consisting of semisolid MS + BA medium followed by transfer to liquid M2 medium supplemented with KIN and IAA, promoted shoot proliferation, differentiation, and rooting simultaneously, even when shoot clusters rather than individual shoots were used as explants. This approach may facilitate large-scale propagation of the species by streamlining the process, increasing production efficiency, and reducing the cultivation period to only 60 d. Furthermore, the total amount of culture medium required throughout the entire process was reduced by approximately 40%.

### 4.2. Percentage of Hyperhydric Explants

A trend toward increased hyperhydricity was observed in liquid media, as previously reported in *A. potatorum* (tobalá agave) [41]. The higher incidence of hyperhydricity observed in liquid MS media may be related to the consistency of the medium, as well as to faster nutrient uptake kinetics due to an ionic imbalance—particularly regarding the availability of ammonium and nitrate in MS medium—which leads to an imbalance in metabolism among cell types and rapid vacuolation of the cells [32]. This relationship became more evident at the end of the second phase, where transferring explants from liquid-to-semisolid medium reduced the percentage of hyperhydric explants. This effect may be attributed to the role of gelling agents as osmotic regulators, modulating water and oxygen availability in the culture medium, in addition to providing physical support to the explants and facilitating nutrient uptake [51,52]. These results are consistent with those reported by Debergh and Maene [53] and Saher et al. [54], supporting the idea that the presence and concentration of agar influence the osmotic potential of the culture system and, consequently, the incidence of hyperhydricity.

### 4.3. Size and Hyperhydricity in a Two-Phase System Under Opposite Medium-Consistency Transitions

In general, medium consistency within the two-phase culture system did not significantly affect cluster growth. Both semisolid and liquid media likely ensured sufficient nutrient availability throughout the culture period; therefore, the explants did not show significant differences in size among treatments. Previous studies have reported that medium consistency can directly influence the availability of nutrients and other medium components through osmotic regulation and the structural properties of the gelling matrix [54,55]. However, under the conditions evaluated in the present study, no evident alterations in overall explant growth were observed. Despite the absence of significant differences in explant size, notable variations in explant morphology were detected. Regarding hyperhydricity, significant differences were associated with the direction of medium consistency transition during the two-phase culture system. The semisolid-to-liquid sequence resulted in a higher percentage of hyperhydric explants than the liquid-to-semisolid transition. The lower incidence of hyperhydricity observed in the latter system may be associated with the initial exposure of explants to greater availability of water, nutrients, and PGRs in liquid medium, as previously reported [56], followed by transfer to semisolid medium, which likely improved regulation of water uptake, enhanced aeration compared with stationary liquid cultures, and promoted better structural tissue development, thereby reducing excessive water accumulation in the explants.

In recent years, liquid culture systems have emerged as an alternative to conventional micropropagation methods based on semisolid media [57]. Consequently, TISs have become one of the most widely used biotechnological tools for the efficient management of liquid media in plant micropropagation [58]. However, excessive hyperhydricity remains a persistent problem, even under bioreactor conditions, and is commonly controlled through immersion and aeration cycles [59]. This response was also reported in *A. guiengola* [31] when different immersion frequencies (6, 12, and 24 h) were evaluated using a twin-container TIS design. Nevertheless, the implementation of TISs involves several limitations, including higher cultivation costs, increased mechanization, greater technical requirements, and a higher risk of contamination. Therefore, reducing these limitations while preserving the advantages of liquid culture represents an important improvement in cultivation efficiency, as demonstrated in the present study through the use of the two-phase culture system without the need for mechanized immersion systems.

### 4.4. Root Production

According to the results, most treatments promoted root development, except for explants cultured in M1 medium supplemented with 5.0 mg L^−1^ BA under both semisolid-to-liquid (T3) and liquid-to-semisolid (T4) transitions, which showed no root formation during either culture phase. This response was likely associated with the effect of BA, since high concentrations of exogenously applied cytokinins have been reported to inhibit or delay root development in some species [60]. Similar results have been documented in *A. parrasana*, where BA concentrations of 0.5–2.0 mg L^−1^ promoted shoot proliferation but slowed rooting [61]. In contrast, explants cultured in M1 medium without PGRs under both semisolid-to-liquid (T1) and liquid-to-semisolid (T2) transitions showed consistent root formation. Several studies have reported that MS medium without added PGRs can promote rooting in *Agave* species [62,63]. Similarly, in *A. guiengola*, MS medium free of PGRs has also been shown to favor root development in propagated shoots [31], which is consistent with the results obtained in the present study regardless of medium consistency. Furthermore, the results suggest a positive effect of medium consistency transition on root formation, particularly when explants were initially cultured in liquid medium and subsequently transferred to semisolid medium. This response was also associated with the lower incidence of hyperhydricity observed under the liquid-to-semisolid system. Similar behavior has been reported in *A. tequilana* [27], where plants cultured in liquid medium developed roots with increased root length, greater thickness, and the formation of adventitious root hairs. This response may be associated with the absence of physical barriers in liquid medium, allowing unrestricted root growth [64], whereas subsequent transfer to semisolid medium may stimulate root hair development and enhance nutrient uptake.

### 4.5. Callus Production

Callogenesis is highly dependent on both the type of explant and the PGRs used [65]. However, it is generally more readily induced in young tissues with high meristematic activity [66], such as the shoot clusters used in the present study. For calli to undergo caulogenesis, high cytokinin concentrations are typically required, often in combination with auxins to stimulate cell division, redifferentiation, and shoot induction. In this context for *A. guiengola*, the basal MS medium with 5.0 mg L^−1^ BA promoted the induction of this morphogenic pathway. Even after 60 d of culture, explants cultured in M1 medium + 5.0 mg L^−1^ BA under both semisolid-to-liquid (T3) and liquid-to-semisolid (T4) transitions maintained active callogenesis and produced multiple adventitious shoots. Similarly, Chávez-Ortíz et al. [31] reported callus formation in *A. guiengola* using different concentrations and combinations of BA and 2,4-D, with the combination of 2.0 and 0.1 mg L^−1^ producing the highest callus induction. Likewise, Vargas [24] achieved indirect caulogenesis using NAA and BA, generating shoots at concentrations of 0.5 mg L^−1^ NAA and 1.5 and 2.0 mg L^−1^ BA. Comparable responses have also been reported in other *Agave* species. In *A. fourcroydes* [63], indirect caulogenesis was induced using 5.0 mg L^−1^ BA in combination with low auxin concentrations of NAA and indole-3-butyric acid (IBA) (0.1 mg L^−1^ each), suggesting that high cytokinin levels together with low auxin concentrations may favor this morphogenic response. In contrast, *A. americana* [67] showed successful indirect caulogenesis with 16.5 mg L^−1^ BA combined with 0.1 mg L^−1^ 2,4-D, indicating considerable genotype-dependent variability among *Agave* species.

## 5. Conclusions

The best conditions for explant growth and development were obtained using the modified medium (M2) supplemented with KIN and IAA at concentrations of 5.0–9.0 mg L^−1^ and 0.1–1.0 mg L^−1^, respectively (T7 and T8). Variations in medium consistency had a stimulatory effect that helped control and reduce excessive hyperhydricity during cultivation, particularly in the liquid-to-semisolid transition system. Hyperhydricity occurred mainly in treatments using M1 medium, especially under liquid culture conditions; however, its incidence decreased after transfer to semisolid medium. Growth and development, and in some cases propagation, occurred through axillary bud proliferation. Organogenic callus formation (caulogenesis) was observed in treatments with M1 medium supplemented with 5.0 mg L^−1^ BA. Root formation was also achieved in explants cultured in M1 medium without BA or in M2 medium. An efficient cultivation system is proposed consisting of two sequential phases: an initial 30 d culture period in semisolid M1 medium supplemented with 5.0 mg L^−1^ BA, followed by a second phase in liquid M2 medium supplemented with KIN and IAA (5.0–9.0 mg L^−1^ and 0.1–1.0 mg L^−1^, respectively) for another 30 d. This system may maximize explant growth and reduce cultivation costs due to the lower volume of culture medium required. Consequently, this approach may reduce resource consumption and overall production costs. This research provides a viable alternative for the in vitro cultivation of *A. guiengola*, improves production efficiency, and contributes valuable information toward understanding the in vitro behavior of this species, thereby supporting future studies focused on its recovery, conservation, and sustainable utilization.

## Figures and Tables

**Table 1:** Treatments.

Treatment	Culture Medium	Regulator	Consistency	
			1–30 d	30–60 d
T1 (control)	M1	-	SS → L	
T2	M1	-	L → SS	
T3	M1	BA (5 mg L^−1^)	SS → L	
T4	M1	BA (5 mg L^−1^)	L → SS	
T5	M2	-	SS → L	
T6	M2	-	L → SS	
T7	M2	KIN + IAA	SS → L	
T8	M2	KIN + IAA	L → SS	

M1 = basal medium MS + L2 vitamins; M2 = modified medium in KNO_3_; NH_4_NO_3_; CaCl_2_·H_2_O; BA = 6-benzylaminopurine; KIN + IAA = formulation of plant growth regulators KIN: 5.0–9.0 mg L^−1^; IAA: 0.1–1.0 mg L^−1^; d = day; SS = semisolid consistency; L = liquid consistency; → = change in consistency.

**Table 2:** Mean explant sizes and percentages of hyperhydricity, root formation, and callus formation in cultured *A. guiengola* Gentry explants.

Treatment	Culture Medium	Regulator	Consistency		Area (cm^2^)		Hyperhydricity (%)		Root Production (%)		Callus Production (%)	
			1–30 d	30–60 d	30 d	60 d	30 d	60 d	30 d	60 d	30 d	60 d
T1 (control)	M1	-	SS → L		1.70 ± 0.25 ^d^	5.0 ± 0.27 ^a^	6.6 ± 6.6 ^b^	33.0 ± 10.43 ^a^	93.2 ± 6.8 ^a^	100 ± 0.0 ^a^	13.2 ± 8.08 ^b^	0.0 ± 0.0 ^c^
T2	M1	-	L → SS		3.02 ± 0.14 ^bc^	2.58 ± 0.12 ^d^	93.2 ± 6.8 ^a^	26.6 ± 19.43 ^a^	46.4 ± 19.93 ^ab^	93.2 ± 6.8 ^a^	39.8 ± 16.35 ^ab^	53.0 ± 13.37 ^ab^
T3	M1	BA (5 mg L^−1^)	SS → L		3.57 ± 0.10 ^ab^	3.32 ± 0.09 ^bcd^	6.6 ± 6.6 ^b^	40.0 ± 24.49 ^a^	0.0 ± 0.0 ^b^	0.0 ± 0.0 ^b^	6.6 ± 6.6 ^b^	59.8 ± 19.44 ^ab^
T4	M1	BA (5 mg L^−1^)	L → SS		3.52 ± 0.18 ^ab^	4.0 ± 0.15 ^b^	100 ± 0.0 ^a^	26.6 ± 19.43 ^a^	0.0 ± 0.0 ^b^	0.0 ± 0.0 ^b^	59.6 ± 12.50 ^a^	93.2 ± 6.8 ^a^
T5	M2	-	SS → L		2.53 ± 0.11 ^cd^	3.0 ± 0.12 ^cd^	0.0 ± 0.0 ^b^	13.2 ± 8.08 ^a^	46.4 ±13.4 ^ab^	13.2 ±13.2 ^b^	0.0 ± 0.0 ^b^	0.0 ± 0.0 ^c^
T6	M2	-	L → SS		3.2 ± 0.37 ^abc^	3.75 ± 0.05 ^bc^	6.6 ± 6.6 ^b^	6.6 ± 6.6 ^a^	93.2 ± 6.8 ^a^	100 ± 0.0 ^a^	13.2 ± 13.2 ^b^	19.8 ± 13.2 ^bc^
T7	M2	KIN + IAA	SS → L		3.67 ± 0.08 ^ab^	5.5 ± 0.21 ^a^	19.8 ± 8.08 ^b^	39.6 ± 12.34 ^a^	26.4 ± 12.34 ^b^	86.4 ± 8.32 ^a^	0.0 ± 0.0 ^b^	26.4 ± 12.34 ^bc^
T8	M2	KIN + IAA	L → SS		4.0 ± 0.15 ^a^	5.0 ± 0.33 ^a^	6.6 ± 6.6 ^b^	0.0 ± 0.0 ^a^	46.4 ±16.98 ^ab^	79.8 ± 13.42 ^a^	0.0 ± 0.0 ^b^	13.2 ± 8.08 ^bc^

Values are mean ± standard error, followed by letters (a,d); different letters indicate significant differences according to Tukey’s HSD test (*p* < 0.05). M1 = basal medium MS + L2 vitamins; M2 = modified medium in KNO_3_; NH_4_NO_3_; CaCl_2_·H_2_O; BA = 6-benzylaminopurine; KIN + IAA = formulation of plant growth regulators kinetin: 5.0–9.0 mg L^−1^; 3-indoleacetic acid: 0.1–1.0 mg L^−1^; d = day; SS = semisolid consistency; L = liquid consistency; → = change in consistency.

**Table 3:** Mean size and percentage of hyperhydricity in a two-phase system under opposite medium-consistency transitions.

Consistency		Area (cm^2^)	Hyperhydricity (%)
1–30 d	30–60 d		
SS → L		4.20 ± 0.25 ^a^	31.45 ± 7.41 ^a^
L → SS		3.83 ± 0.21 ^a^	10.47 ± 5.71 ^b^

Values are expressed as mean ± standard error, followed by letters (a, b); different letters indicate significant differences according to Tukey’s HSD test (*p* < 0.05). d = day; SS = semisolid consistency; L = liquid consistency; → = change in consistency.

## Data Availability

The original contributions presented in this study are included in the article. Further inquiries can be directed to the corresponding author.

## References

[1] Moreno-Martíez K.S., Monja-Mio K.M. (2021). Cultivo in vitro de agaves. Rev. Cienc..

[2] García-Mendoza A.J., Linares L., Dávila P., Chiang F., Bye R., Elias T. (1995). Riqueza y endemismo de la familia de las Agavaceae en México. Conservación de Plantas en Peligro de Extinción: Diferentes Enfoques.

[3] García-Mendoza A.J. (2007). Los agaves de México. Ciencias.

[4] Nava-Cruz N.Y., Medina-Morales M.A., Martinez J.L., Rodriguez R., Aguilar C.N. (2015). *Agave* biotechnology: An overview. Crit. Rev. Biotechnol..

[5] Davis S.C., Ortiz-Cano H.G. (2023). Lessons from the history of *Agave*: Ecological and cultural context for valuation of CAM. Ann. Bot..

[6] García-Mendoza A.J. (2003). Agave guiengola.

[7] SEMARNAT (2019). Modificación del Anexo Normativo III, Lista de Especies en Riesgo de la Norma Oficial Mexicana NOM-059-SEMARNAT-2010:101.

[8] García-Mendoza A.J., Sandoval-Gutiérrez D. *Agave guiengola*, The IUCN Red List of Threatened Species 2019: e.T22486574A22486582. https://dx.doi.org/10.2305/IUCN.UK.2019-3.RLTS.T22486574A22486582.en.

[9] Rodríguez S.P., Muñoz A., Durán R., Hernández J., Limón J.P., Hernández-Hernández H.M. (2021). Micropropagación como alternativa para evitar la extinción de plantas endémicas. J. Bioeng. Biomed. Res..

[10] Groenewald E.G., Wessels D.C.J., Koeleman A. (1977). Callus formation and subsequent plant regeneration from seed tissue of an *Agave* species (Agavaceae). Z. Pflanzenphysiol..

[11] Robert M.L., Herrera J.L., Contreras F., Scorer K.N. (1987). In vitro propagation of *Agave fourcroydes* Lem. (Henequén). Plant Cell Tissue Organ Cult..

[12] Powers D.E., Backhaus R.A. (1989). In vitro propagation of *Agave arizonica* Gentry & Weber. Plant Cell Tissue Organ Cult..

[13] Hazra S.K., Das S., Das A.K. (2002). Sisal Plant regeneration via organogenesis. Plant Cell Tissue Organ Cult..

[14] Valenzuela-Sánchez K.K., Juárez-Hernández R.E., Cruz-Hernández A., Olalde-Portugal V., Valverde M.E., Paredes-López O. (2006). Plant regeneration of *Agave tequilana* by indirect organogenesis. In Vitro Cell. Dev. Biol. Plant.

[15] Portillo L., Santacruz-Ruvalcaba F., Gutiérrez-Mora A., Rodríguez-Garay B. (2007). Somatic embryogenesis in *Agave tequilana* Weber cultivar azul. In Vitro Cell. Dev. Biol. Plant.

[16] Silos-Espino H., González-Cortés N., Carrillo-López A., Guevara-Lara F., Valverde-González M.E., Paredes-López O. (2007). Chemical composition and in vitro propagation of *Agave salmiana* “Gentry”. J. Hortic. Sci. Biotechnol..

[17] Rosales M.S., Alpuche A.G., Vasco N.L., Pérez-Molphe-Balch E. (2008). Efecto de las citoquininas en la propagación in vitro de agaves mexicanos. Rev. Fitotec. Mex..

[18] Cheng Y., Chen X., Hu F., Yang H., Yue L., Trigiano R.N., Cheng Z.M. (2014). Micropropagation of *Agave americana*. HortScience.

[19] Sánchez-Mendoza E.A., Pérez-Molphe-Balch E., Guzmán-Mendoza R., Ruiz-Aguilar G., García-Munguía A.M., Costilla-Salazar R., Núñez-Palenius H.G. (2025). Plant Growth Regulators Use in the In Vitro Culture of *Agave* Species. Plants.

[20] Santacruz-Ruvalcaba F., Castañeda-Nava J.J., Villanueva-Gónzalez J.P., García-Sahagún M.L., Portillo L., Contreras-Pacheco M.L. (2022). Micropropagación de *Agave maximiliana* Baker por proliferación de yemas axilares. Polibotánica.

[21] de la Cruz-Olvera A., Serrano M., Rayas A.A., García R. (2025). Micropropagación de *Agave angustifolia* Haw., con fines de aprovechamiento y conservación. Rev. Mex. Cienc. For..

[22] Aguilar-Jiménez D., Mendez-Castillo G.K., Reyes-Trejo B., Valadez-Moctezuma E., Rodríguez-De la O J.L., Ramírez-Mosqueda M.A., Cadena-Zamudio J.D., Aguirre-Noyola J.L. (2026). Conservación in vitro de *Agave potatorum* Zucc y *Agave marmorata* Roezl. Conservation of Plant Genetic Resouces.

[23] Rescalvo-Morales A., Benítez-Zárate J., Ojeda G., Herrera-Alamillo M.Á., Sánchez-Teyer L.F., Monja-Mio K.M. (2026). Influence of mother plant age on the in vitro propagation of *Agave cupreata* Trel. & A. Berger via organogenesis. Discov. Plants.

[24] Vargas A. (2017). Micropropagación de *Agave guiengola* Gentry, una Especie Endémica en Peligro de Extinción del Estado de Oaxaca, México. Bachelor’s Thesis.

[25] Bello-Bello J.J., Mancilla-Álvarez E., Spinoso-Castillo J.L. (2025). Scaling-up procedures and factors for mass micropropagation using temporary immersion systems. In Vitro Cell. Dev. Biol. Plant.

[26] Monja-Mio K.M., Barredo F., Herrera G., Esqueda M., Robert M.L. (2015). Development of the stomatal complex and leaf surface of *Agave angustifolia* Haw. ‘Bacanora’ plantlets during the in vitro to ex vitro transition process. Sci. Hortic..

[27] Monja-Mio K.M., Olvera-Casanova D., Herrera-Herrera G., Herrera-Alamillo M.A., Sánchez-Teyer F.L., Robert M.L. (2020). Improving of rooting and ex vitro acclimatization phase of *Agave tequilana* by temporary immersion system (BioMINT™). In Vitro Cell. Dev. Biol. Plant.

[28] Ortiz-Mena M.I., Gutiérrez-Mora A., Vega-Ramos K.L., Rodríguez-Domínguez J.M., Tapia-Campos E. (2023). Micropropagación de *Agave* spp. mediante proliferación de yemas axilares en sistemas de inmersión temporal. Los agaves y Sus Derivados: Tendencias Científicas, Uso Sostenible y Patrimonio.

[29] Mancilla-Álvarez E., Spinoso-Castillo J.L., Muñoz-Márquez T.R.A., Palacios-Pardo K.F., Bello-Bello J.J. (2024). Temporary immersion bioreactor as an efficient method for in vitro propagation of *Agave marmorata*. S. Afr. J. Bot..

[30] Mancilla-Álvarez E., Spinoso-Castillo J.L., Schettino-Salomón S.S., Bello-Bello J.J. (2024). Temporary immersion systems induce photomixotrophism during in vitro propagation of agave Tobalá. 3 Biotech.

[31] Chávez-Ortíz L.I., Morales-Domínguez J.F., Rodríguez-Sahagún A., Pérez-Molphe-Balch E. (2021). In vitro propagation of *Agave guiengola* Gentry using semisolid medium and temporary immersion bioreactors. Phyton.

[32] Murashige T., Skoog F. (1962). A revised medium for rapid growth and bioassays with tobacco tissue cultures. Physiol. Plant.

[33] del Carmen Ríos-Ramírez S., Enríquez-del Valle J.R., Ortiz G.R., Ruíz-Luna J. (2017). Benzylaminopurine and indol-3-acetic acid concentrations in in vitro proliferation of *Agave angustifolia* adventitious shoots. Int. J. Agric. Nat. Resour..

[34] Arzate-Fernández A.M., Martínez-Velasco I., Alvarez-Aragón C., Martinez-Martinez S.Y., Norman-Mondragon T.H. (2020). Morphogenetic response of two *Agave* species regenerated in vitro. Trop. Subtrop. Agroecosyst..

[35] Puente-Garza C.A., Gutiérrez-Mora A., García-Lara S. (2015). Micropropagation of *Agave salmiana*: Means to production of antioxidant and bioactive principles. Front. Plant Sci..

[36] Phillips G.C., Collins G.B. (1979). In vitro tissue culture of selected legumes and plant regeneration from callus cultures of red clover. Crop. Sci..

[37] Alcántara J.S., Acero J., Alcántara J.D., Sánchez R.M. (2019). Main hormonal regulators and their interactions in plant growth. Nova.

[38] García H.T., Benavides A., Escobedo L., Villareal J.A., Cornejo E. (2011). Hyperhydricity control of in vitro shoots of *Turbinicarpus valdezianus* (Moller), G & F. Phyton-Int. J. Exp. Bot..

[39] Savio L.E.G., Astarita L.V., Santarém E.R. (2012). Secondary metabolism in micropropagated *Hypericum perforatum* L. grown in non-aerated liquid medium. Plant Cell Tissue Organ Cult..

[40] Myeong-Jin L., Jong-Eun H., Hosakatte N.M., Hyun-Young S., Su-Young L., So-Young P. (2024). A Comparison of semi-solid, liquid, and temporary immersion bioreactor systems for effective plant regeneration of *Gerbera jamesonii* “Shy Pink”. Horticulturae.

[41] Correa-Hernández L., Baltazar-Bernal O., Sánchez-Páez R., Bello-Bello J.J. (2022). In vitro multiplication of agave tobala (*Agave potatorum* Zucc.) using Ebb-and-Flow bioreactor. S. Afr. J. Bot..

[42] Şen A. (2012). Oxidative stress studies in plant tissue culture. Antioxidant Enzyme.

[43] Roberts A.V., Smith E.F. (1990). The preparation in vitro of *Chrysanthemum* for transplantation to soil. I. Protection of roots by cellulose plugs. Plant Cell Tissue Organ Cult..

[44] del Rivero N., Quiala E., Agramante D., Barbón R., Camacho W., Morejón L., Pérez M. (2004). Empleo de sistemas de inmersión temporal para la multiplicación in vitro de brotes de *Anthurium andraeanum* Lind. var. Lambada. Biotecnol. Veg..

[45] Hempfling T., Preil W., Hvoslef-Eide A.K., Preil W. (2005). Application of a temporary immersion system in mass propagation of *Phalaenopsis*. Liquid Culture Systems for In Vitro Plant Propagation.

[46] Dévora-Rodríguez D.G., Pulido-Díaz C., Chávez-Simental J.A., Ortiz-Sánchez I.A., Loera-Gallegos H.M., Prieto-Ruiz J.A. (2021). Reguladores de crecimiento en el desarrollo vegetativo de vitroplantas de *Agave durangensis* Gentry. Ecosistemas Recur. Agropecu..

[47] Reyes-Silva A.I., Nuñez-Palenius H.G., Ocampo G., Pérez-Molphe-Balch E. (2022). Regeneración in vitro de *Agave wocomahi* Gentry (Asparagaceae). Rev. Fitotec. Mex..

[48] Castillo-Martínez C.R., Velasco-Bautista E., Betanzos-Jiménez R.P., Aragón-Cuevas F. (2023). Mass propagation of tobala mezcal maguey (*Agave potatorum* Zucc.) in a temporary immersion system compared with a solid medium. Agro Product..

[49] Duarte-Aké F., De-la-Peña C. (2021). High cytokinin concentration and nutrient starvation trigger DNA methylation changes in somaclonal variants of *Agave angustifolia* Haw. Ind. Crops Prod..

[50] Spinoso-Castillo J.L., Pérez-Sato J.A., Schettino-Salomón S.S., Bello-Bello J.J. (2022). An alternative method for medium-term in vitro conservation of different plant species through gibberellin inhibitors. In Vitro Cell. Dev. Biol.-Plant.

[51] Martin D.A., Cárdenas O., Constantino J. (2012). Sustancias utilizadas como agente gelificante alternativas al agar en medios de cultivo para propagación in vitro. Rev. Investig. Agrar. Ambient.

[52] López-Escamilla A.L. (2016). Efecto de diferentes agentes gelificantes en la germinación y desarrollo in vitro de plántulas de *Echinocactus platyacanthus* Link et Otto (Cactaceae). Polibotánica.

[53] Debergh P.C., Maene L.J. (1981). A scheme for commercial propagation of ornamental plants by tissue culture. HortScience.

[54] Saher S., Piqueras A., Hellin E., Olmos E. (2004). Hyperhydricity in micropropagated carnation shoots: The role of oxidative stress. Physiol. Plant.

[55] Bošnjak D., Marković M., Agić D., Vinković T., Tkalec M., Ravnjak B., Stanisavljević A. (2021). The influence of nutrient media modification on the morphological parameters in raspberry (*Rubus idaeus* L.) micropropagation in the liquid and semi-solid media. Poljoprivreda.

[56] Mirzabe A.H., Hajiahmad A., Fadav A., Rafiee S. (2022). Temporary immersion systems (TISs): A comprehensive review. J. Biotechnol..

[57] Méndez-Hernández H.A., Loyola-Vargas V.M. (2024). Plant micropropagation and temporary immersion systems. Plant Cell Culture Protocols.

[58] Bello-Bello J.J., Cruz-Cruz C.A., Pérez-Guerra J.C. (2019). A new temporary immersion system for commercial micropropagation of banana (*Musa* AAA cv. Grand Naine). In Vitro Cell. Dev. Biol. Plant.

[59] Steinmacher D.A., Guerra M.P., Saare-Surminski K., Lieberei R. (2011). A temporary immersion system improves in vitro regeneration of peach palm through secondary somatic embryogenesis. Ann. Bot..

[60] Jing H., Strader L.C. (2019). Interplay of auxin and cytokinin in lateral root development. Int. J. Mol. Sci..

[61] Santacruz-Ruvalcaba F., Gutiérrez-Pulido H., Rodríguez-Garay B. (1999). Efficient in vitro propagation of *Agave parrasana* Berger. Plant Cell Tissue Organ Cult..

[62] Martínez-Palacios A., Ortega-Larrocea M.P., Chávez V.M., Bye R. (2003). Somatic embryogenesis and organogenesis of *Agave victoriae-reginae*: Considerations for its conservation. Plant Cell Tissue Organ Cult..

[63] Zhang Y.-M., Li X., Chen Z., Li J.-F., Lu J.-Y., Zhou W.-Z. (2013). Shoot organogenesis and plant regeneration in *Agave* hybrid, No. 11648. Sci. Hortic..

[64] Ayenew B., Tadesse T., Gebremariam E., Mengesha A., Tefera W. (2013). Efficient use of temporary immersion bioreactor (TIB) on pineapple (*Ananas comosus* L.) multiplication and rooting ability. J. Microbiol. Biotechnol. Food Sci..

[65] Rodríguez M.M., Latsague M.I., Chacón M.A., Astorga P.K. (2014). In vitro induction of callogenesis and indirect organogenesis from explants of cotyledon, hypocotyl and leaf in *Ugni molinae*. Bosque.

[66] Varshney A., Sangapillai R., Patil M.S., Johnson T.S. (2011). Histological evidence of morphogenesis from various explants of *Jatropha curcas* L.. Trees.

[67] Lecona-Guzmán C.A., Reyes-Zambrano S., Barredo-Pool F.A., Abud-Archila M., Montes-Molina J.A., Rincón-Rosales R., Gutierrez-Miceli F.A. (2017). In vitro propagation of *Agave americana* by indirect organogenesis. HortScience.

